# The clinical value of Serum hyaluronic acid, procollagen III, N-terminal propeptide levels sST2 and cfDNA in predicting the myocardial damage in children with severe pneumonia

**DOI:** 10.5937/jomb0-51223

**Published:** 2025-01-24

**Authors:** Jia Haoran, Liu Ye, Zhao Tingting, Wang Dexing, Du Meng, Wang Weiwei

**Affiliations:** 1 Baoding Hospital, Beijing Children's Hospital Affiliated to Capital Medical University, Neonatology Department, Baoding, China; 2 Baoding Hospital, Beijing Children's Hospital Affiliated to Capital Medical University, Pediatric Department, Baoding China

**Keywords:** serum hyaluronic acid, procollagen III, NTproBNP, sST2, cfDNA, guiding evidence-based nursing, clinical value, serumska hijaluronska kiselina, prokolagen III, NT-proBNP sST2, cfDNA, nega bolesnika zasnovana na dokazima, klinička vrednost

## Abstract

**Background:**

Severe pneumonia complicated by myocardial damage is a serious condition in children, and early prediction and intervention are crucial for improving outcomes. To explore the clinical value of serum hyaluronic acid, procollagen III N-terminal propeptide, sST2, and cfDNA levels in predicting myocardial damage in children with severe pneumonia.

**Methods:**

A case-control study was conducted on 140 children with severe pneumonia, divided into a control group (n=70) and a group with disease complicated by myocardial injury (n=70). Biomarker levels (hyaluronic acid, procollagen III, N-terminal propeptide levels, sST2, and cfDNA) were detected through biochemical analysis, and clinical outcomes were compared between the two groups.

**Results:**

Logistic regression analysis identified hypoalbuminaemia, age <3 years, and elevated levels of serum hyaluronic acid, procollagen III N-terminal propeptide, sST2, and cfDNA as independent risk factors for myocardial damage. The AUC of these biomarkers in predicting myocardial injury ranged from 0.68 to 0.78, with sensitivities and specificities of 65-80%. The combined prediction AUC was 0.92, with a sensitivity of 87.6% and a specificity of 77%. The control group had lower serum NT-proBNP, CK, CK-MB, and C-reactive protein levels and better clinical outcomes than the myocardial injury group.

**Conclusions:**

Serum hyaluronic acid, procollagen III, NTproBNP, sST2, and cfDNA significantly predict severe pneumonia complicated by myocardial damage. Nursing strategies based on these biomarkers can effectively improve treatment outcomes, demonstrating significant clinical application value.

## Abstract (sr)

**Uvod:**

Teška pneumonija komplikovana oštećenjem miokarda je ozbiljno stanje kod dece, a rana predikcija i intervencija ključne su za poboljšanje ishoda. Cilj je bio da se istraži klinička vrednost nivoa serumske hijaluronske kiseline, N-terminalnog propeptida prokolagena III, sST2 i cfDNA u predviđanju oštećenja miokarda kod dece sa teškom pneumonijom.

**Metode:**

Sprovedena je studija slučaja sa kontrolnom grupom na 140 dece sa teškom pneumonijom, koja su bila podeljena u kontrolnu grupu (n=70) i grupu sa bolešću komplikovanom oštećenjem miokarda (n=70). Nivoi biomarkera (hijaluronska kiselina, prokolagen III, N-terminalni propeptid, sST2 i cfDNA) određeni su biohemijskom analizom, a klinički ishodi su upoređeni između dve grupe.

## Introduction

Severe pneumonia complicated with myocardial damage is a serious condition in children that requires early identification and effective monitoring to improve prognosis and reduce mortality. Traditional diagnostic methods have limitations, but using biomarkers such as NT-proBNP and cardiac troponin has shown promise in improving the diagnosis and management of myocardial damage in children [1] [2] [3]. Mycoplasma Pneumoniae is most knowingly associated with myocardial damage in children [4], while it also has been observed in viral pneumonia-like COVID-19 in children [1].

However, for children with severe pneumonia complicated with myocardial damage, more specific and sensitive biomarkers are needed to guide treatment and nursing strategies. Previously, D-dimer, a marker of coagulation status, was found to be a strong predictor of severe Mycoplasma Pneumoniae pneumonia (SMPP) in children, with levels above 308 μg/L indicating a higher risk of complications such as pleural effusion, myocardial and liver damage [5]. However, other biomarkers with more specificity for myocardial damage might be helpful, and we have explored some of them. As a polysaccharide, hyaluronic acid is involved in many biological processes, including cell multiplication, migration and inflammation, and its role in myocardial injury has been widely discussed in recent years [6]. Procollagen III is an essential sign of collagen synthesis, which can reflect the repair process of myocardial cells and the occurrence of fibrosis [7]. As a response molecule to cardiac stress, sST2 has been proven to have prognostic value in heart failure [8] [9]. As a sign of apoptosis and tissue injury, the increase in cfDNA level may directly reflect the degree of myocardial cell injury [10]. Through the comprehensive analysis of these biomarkers, this study aims to evaluate their clinical value in children with severe pneumonia complicated with myocardial damage and to explore their application potential in disease monitoring, curative effect evaluation and prognosis judgment. We assume that the levels of these biomarkers are closely related to the severity of myocardial damage. They can be used as important indicators to evaluate the effect of nursing strategies to provide more accurate and personalized treatment and nursing programs for children with severe pneumonia complicated with myocardial damage.

## Materials and methods

### General information

One hundred forty children with severe pneumonia complicated with myocardial damage admitted to our hospital from May 2022 to April 2024 were recruited as the research subjects. All patients were informed of the protocol and signed informed consent forms when included in the study.

Inclusion criteria: Children are between 1 and 8 years old, regardless of sex; diagnosed with severe pneumonia through imaging examination and laboratory data analysis. All children needed to evaluate whether there was myocardial damage by cardiac biochemical markers (such as cardiac troponin, NT-proBNP, etc.) and echocardiography [11]; The legal guardian of the child provided written informed consent.

Exclusion criteria: children with known heart illness, such as congenital heart illness and rheumatic heart illness; Children with major chronic illness, such as chronic nephropathy, chronic liver disease and metabolic diseases. Children with severe infections in other parts; Children with recent (such as in the past month) major surgical history; Children who were receiving drugs that may affect the evaluation of myocardial function (such as some cardiovascular drugs) [11].

Medical ethics issues: This study was approved by our hospital’s relevant ethics review committee, and then informed consent was provided for the scheme when it was included in the research object, and all procedures complied with ethical norms and regulations.

### Methods

### Observation indicators

### (1) ELISA detection of serum hyaluronic acid, procollagen III, N-terminal peptide, sST2, cfDNA, NT proBNP, and C-reactive protein levels

About 3 mL of blood was drawn from the patient’s vein and placed in a test tube containing coagulant. The blood was naturally coagulated at room temperature for 30 minutes. Subsequently, the serum was separated by centrifugation. The levels of serum hyaluronic acid, procollagen III, N-terminal propeptide, sST2, cfDNA, NT-proBNP and C-reactive protein were quantitatively analyzed by using a special ELISA kit.

### (2) Determination of CK and CK-MB levels by enzyme activity method.

After obtaining venous blood, the serum was separated. Enzyme activity was measured. Serum samples were added to the reaction solution containing specific substrates and incubated at 37°C for a certain period of time (for example, 5 minutes). The substrate was catalyzed by CK or CK-MB to produce a colour change, and the absorbance change after the reaction was determined. According to the degree and rate of colour change, CK and CK-MB activity levels were calculated.

### (3) Clinical treatment effect analysis

Before the start of treatment, the two groups of patients were evaluated in detail, including vital signs (heart rate, blood pressure), laboratory indicators (blood, urine test), imaging examination (X-ray or MRI) and so on, to ensure that baseline data were obtained. Define the therapeutic effect standard: it is divided into obvious effect, effective and ineffective. Remarkable effect: the symptoms disappeared or improved by more than 75%. Effective: the degree of symptom improvement is between 50% and 75%. Ineffective: the degree of improvement in symptoms is less than 50%, or there is no improvement. After the treatment, the patients were evaluated comprehensively, including the previously defined indicators, to evaluate the treatment effect.

### Statistical analysis

This study used SPSS20.0 statistical software. The measurement data is represented by »mean ± standard deviation« (x̄±s), inter-group comparisons are performed using one-way ANOVA, and intergroup comparisons are performed using t-test. The counting data is expressed as a percentage (%), and inter-group comparisons are made using χ^2^ analysis. *P*<0.05 represents a statistically significant difference.

## Results

### General data analysis of children

According to the general data of children, there was no distinction between the two groups (P>0.05) (Table 1).

**Table 1:** General data analysis of children.

Project	Group with myocardial<br>damage (n=70)	Group without myocardial<br>damage (n=70)	T value /χ^2^<br>value	P value
Gender (male: female)	35 35	32 38	3.001	0.552
Age (years)	5.18±2.44	5.75±2.19	5.674	0.417
Height	98.47±5.66	95.18±4.57	5.288	0.266
Weight	19.01±2.35	18.49±2.22	2.017	0.519
BMI (kg/m^2^)	12.33±2.02	12.77±2.61	4.253	0.684
Nutritional status (good/poor)	68:2	65:5	2.991	0.229
Hyaluronic acid (μg/L)	116.47±12.39	105.25±8.44	1.246	0.222
Procollagen III (ng/mL)	16.21±2.56	15.34±2.77	1.307	0.116
N-terminal prepeptide (ng/mL)	0.47±0.16	0.45±0.14	1.112	0.137
sST2 (ng/mL)	36.49±4.15	35.83±5.37	2.648	0.101
cfDNA (ng/mL)	584.41±22.69	579.97±28.65	1.553	0.234

### Logistic regression analysis of risk factors

Logistic regression analysis showed that hypoproteinemia, age < 3 years, serum hyaluronic acid, procollagen III, N-terminal propeptide, sST2 and cfDNA were independent risk factors of myocardial damage in children with severe pneumonia (P<0.05) (Table 2).

**Table 2:** Logistic factor analysis of severe pneumonia complicated with myocardial damage.

Variable	Beta coefficient	Standard error	OR	P value
Hypoproteinemia	1.22	0.41	2.96	0.01
Age < 3 years old	0.86	0.35	3.18	0.04
hyaluronic acid	1.59	0.54	4.47	0.03
Procollagen III	1.13	0.29	3.05	0.01
N-terminal propeptide (ng/mL)	0.96	0.17	3.76	0.03
sST2 (ng/mL)	1.36	0.44	2.04	0.01
cfDNA (ng/mL)	1.45	0.21	1.55	0.02

### ROC analysis of predictive efficacy of biomarkers

**Figure 1:**
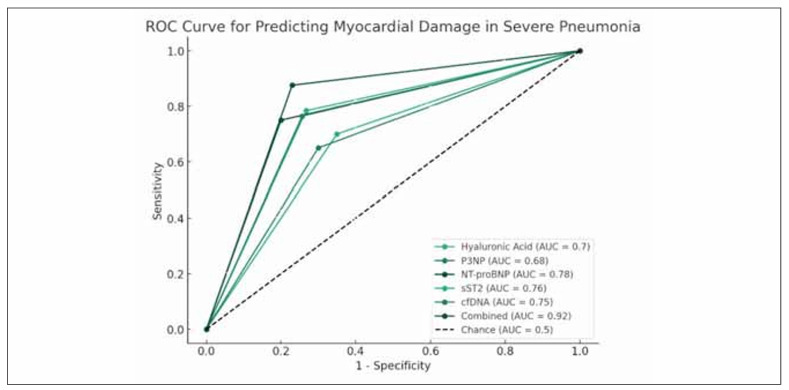
ROC curve of biomarkers in predicting severe pneumonia complicated with myocardial damage.

According to the ROC curve, the efficacy of biomarkers in predicting myocardial damage in children with severe pneumonia was analyzed. The AUC, sensitivity and specificity of serum hyaluronic acid in predicting myocardial damage in children with severe pneumonia were 0.70, 70% and 65%, respectively. The predictive AUC of procollagen III was 0.68, with a sensitivity of 65% and a specificity of 70%. The predicted AUC of NT-proBNP was 0.78, with a sensitivity of 75% and a specificity of 80%. The predicted AUC of sST2 was 0.76, with a sensitivity of 78.43% and a specificity of 73.25%. The predicted AUC of cfDNA was 0.75, with a sensitivity of 76.49% and a specificity of 74.31%. The joint prediction AUC was 0.92, the sensitivity was 87.60%, and the specificity was 77% (Figure 1, Table 3).

**Table 3:** ROC Analysis of Predictive Efficiency of Biomarkers.

Biomarker	Sensitivity	Specificity	AUC
Hyaluronic acid	70%	65%	0.70
Procollagen III	65%	70%	0.68
N-terminal propeptide (ng/mL)	72%	68%	0.72
sST2 ng/mL	78%	73%	0.76
cfDNA ng/mL	76%	74%	0.75
Joint forecast	88%	85%	0.92

### Comparison of markers of myocardial injury

The levels of serum NT-proBNP, CK, CK-MB and C-reactive protein in the control group were lower than those in the control group (P<0.05) (Table 4).

**Table 4:** Comparison of myocardial injury markers.

Markers	Control group (n=70)	Control group (n=70)	T value	P value
NT-proBNP pg/mL	1396.35±33.58	1985.22±41.24	15.209	0.013
CK U/L	177.39±15.64	246.32±18.35	13.667	0.026
CK-MB U/L	22.63±3.51	37.19±4.38	11.284	0.004
C-reactive protein (mg/L)	12.52±1.18	20.36±2.55	9.215	0.003

### Comparison of improvement of clinical symptoms

After implementing nursing interventions, the control group showed an increase in clinical symptom efficacy and effectiveness compared to the control group (*P*<0.05), while the ineffective rate decreased compared to the control group (*P*<0.05) (Table 5).

**Table 5:** Comparison of Clinical Symptoms Improvement.

Index	Control group (n=70)	Control group (n=70)	χ^2^ value	* P value *
Remarkable effect (%)	56 80.00%	42 60.00%	9.237	0.026
Effective (%)	63 90.00%	53 75.71%	14.162	0.003
Invalid (%)	8 11.43%	17 24.29%	13.885	0.012

## Discussion

This study explored the clinical value of serum biomarkers such as hyaluronic acid, procollagen III, N-terminal propeptide, sST2 and cfDNA in detecting children with severe pneumonia complicated with myocardial damage. The results showed that the level of these biomarkers in patients with myocardial damage was significantly higher than in patients without myocardial damage, indicating a significant correlation between these biomarkers and myocardial damage.

Qi et al. [4] explored the changes in myocardial enzymes, including AST, CK, CK-MB, and LDH, in children with Mycoplasma pneumoniae pneumonia (MPP) and found that these enzymes were elevated during the acute phase of the disease, correlating with fever, fever duration, and extrapulmonary organ damage. Although both studies investigate the relationship between biomarkers and myocardial damage in pediatric patients with pneumonia, our study and that of Qi et al. [4] differ in focus and findings. Specifically, our study examined the clinical value of serum biomarkers such as hyaluronic acid, procollagen III N-terminal propeptide, sST2, and cfDNA. Our results indicate that these biomarkers were significantly elevated in patients with myocardial damage, suggesting a correlation between these biomarkers and myocardial injury. Notably, while Qi et al. [4] focused on the diagnostic value of myocardial enzymes in MPP, our study emphasizes the potential of serum biomarkers in guiding nursing strategies for children with severe pneumonia complicated by myocardial damage. Future studies could explore the combined use of these biomarkers to improve diagnosis and treatment outcomes in pediatric patients with pneumonia.

Our study and the study by Lai et al. [11] share some similarities in their findings. Lai et al. [11] found that high-sensitivity C-reactive protein (hs-CRP) was a valuable index for evaluating myocardial damage and prognosis in children with mycoplasmal pneumonia, and that its combination with creatine kinase isoenzyme (CK-MB) detection had guiding significance for monitoring and treatment. Similarly, our study found that serum biomarkers such as hyaluronic acid, procollagen III, N-terminal propeptide, sST2, and cfDNA were significantly higher in patients with myocardial damage, indicating a correlation between these biomarkers and myocardial damage. However, our study focused on a broader range of biomarkers and their potential in guiding nursing strategies for children with severe pneumonia complicated with myocardial damage. In contrast, Lai et al. [11] focused on the specific role of hs-CRP in evaluating myocardial damage and prognosis. The findings of both studies suggest that biomarkers can play a crucial role in diagnosing and managing myocardial damage in pediatric patients with pneumonia and highlight the need for further research to explore the clinical utility of these biomarkers in different clinical settings.

While our study focused on the clinical value of serum biomarkers in detecting children with severe pneumonia complicated by myocardial damage, the case reports by Li et al. [12] highlighted the importance of emergency treatment and nursing in children with severe pneumonia complicated by heart failure and respiratory failure. The case reports emphasized the need for intensive care, including closely monitoring disease progression, rational oxygen therapy, and supporting vital organs to prevent complications and reduce mortality.

While our study focused on the clinical value of serum biomarkers, including cell-free DNA (cfDNA), in detecting children with severe pneumonia complicated with myocardial damage, the study by Ngo et al. [13] investigated the role of platelet factor 4 (PF4) in limiting the thrombogenicity and endothelial injury induced by neutrophil extracellular traps (NETs) and cfDNA. The study found that PF4 interacts with cfDNA and NETs to prevent their cleavage to short-fragment and single-stranded cfDNA, which can activate the contact pathway of coagulation and promote thrombosis. In contrast, our study demonstrated that cfDNA is a valuable biomarker for detecting myocardial damage in children with severe pneumonia. However, the findings of Ngo et al.’s [13] study suggest that the thrombogenic potential of cfDNA may be mitigated by PF4, which could have implications for treating sepsis and other conditions characterized by elevated levels of cfDNA.

Scott et al. [14] investigated the relationship between total cell-free DNA (TCF) and clinical outcomes after heart transplantation in pediatric and adult patients. The study found that elevated TCF levels were associated with increased mortality and treatment for infection. In contrast, increased donor fraction (DF) levels were associated with rejection and cardiac allograft vasculopathy. In contrast, our study demonstrated that cfDNA is a valuable biomarker for detecting myocardial damage in children with severe pneumonia. However, the findings of Scott et al. [14] suggest that TCF may be a more sensitive and specific biomarker for predicting mortality and infection in the context of heart transplantation. Furthermore, the observation that TCF and DF levels can inform treatment after heart transplantation highlights the potential utility of these biomarkers in guiding clinical decision-making. Our study found that cfDNA was significantly higher in patients with myocardial damage, which is consistent with the idea that cfDNA may be a marker of tissue injury and inflammation.

In a word, the results of this study show that serum hyaluronic acid, procollagen III, NT-proBNP, sST2 and cfDNA, as biomarkers, have significant value in predicting severe pneumonia complicated with myocardial damage. The nursing strategy based on these biomarkers can effectively improve the treatment effect and show important clinical application value.

## Dodatak

### Acknowledgements

None.

### Fundings

Beijing Children’s Hospital, affiliated with Capital Medical University, Baoding Hospital, supports the research. The value of serum sST2 and cfDNA in predicting myocardial damage in children with severe pneumonia,(No.2341ZF390).

### Ethical consideration

This study was conducted following the principles of the Declaration of Helsinki and was approved by the Ethics Committee of Beijing Children’s Hospital, Capital Medical University (approval number: (No.2341ZF390)). Informed consent was obtained from the parents or legal guardians of all children enrolled in the study. The study protocol was designed to minimize risk and ensure the privacy and confidentiality of all participants. All data were anonymized and de-identified to protect the privacy of the participants.

### Author contribution

HJ and YL conceived and designed the study. HJ, YL, and TTZ collected and analyzed the data. DW and WW provided clinical expertise and guidance. MD assisted with data interpretation and manuscript preparation. All authors contributed to the writing and revision of the manuscript. HJ and YL are responsible for the final version of the manuscript.

### Conflict of interest statement

All the authors declare that they have no conflict of interest in this work.
